# Cannabidiol and fluorinated derivative anti-cancer properties against glioblastoma multiforme cell lines, and synergy with imidazotetrazine agents

**DOI:** 10.1038/s44276-024-00088-0

**Published:** 2024-09-09

**Authors:** Alice Brookes, Nicholas Kindon, David J. Scurr, Morgan R. Alexander, Pavel Gershkovich, Tracey D. Bradshaw

**Affiliations:** School of Pharmacy, University of Nottingham, Nottingham, NG7 2RD UK

## Abstract

**Background:**

Glioblastoma multiforme (GBM) is an aggressive cancer with poor prognosis, partly due to resistance to the standard chemotherapy treatment, temozolomide (TMZ). Phytocannabinoid cannabidiol (CBD) has exhibited anti-cancer effects against GBM, however, CBD’s ability to overcome common resistance mechanisms to TMZ have not yet been investigated. 4’-Fluoro-cannabidiol (4’-F-CBD, or HUF-101/PECS-101) is a derivative of CBD, that exhibits increased activity compared to CBD during in vivo behavioural studies.

**Methods:**

This anti-cancer activity of cannabinoids against GBM cells sensitive to and representing major resistance mechanisms to TMZ was investigated. Cannabinoids were also studied in combination with imidazotetrazine agents, and advanced mass spectrometry with the 3D OrbiSIMS was used to investigate the mechanism of action of CBD.

**Results:**

CBD and 4’-F-CBD were found to overcome two major resistance mechanisms (methylguanine DNA-methyltransferase (MGMT) overexpression and DNA mismatch repair (MMR)-deficiency). Synergistic responses were observed when cells were exposed to cannabinoids and imidazotetrazine agents. Synergy increased with T25 and 4’-F-CBD. 3D OrbiSIMS analysis highlighted the presence of methylated-DNA, a previously unknown anti-cancer mechanism of action of CBD.

**Conclusions:**

This work demonstrates the anti-cancer activity of 4’-F-CBD and the synergy of cannabinoids with imidazotetrazine agents for the first time and expands understanding of CBD mechanism of action.

## Background

It has been reported that cannabinoids exhibit anti-cancer properties [1–3]. Most activity of cannabinoids is considered to be a result of interaction with cannabinoid receptors 1 and 2 (CB1 and CB2) of the endocannabinoid system. It has been demonstrated that CB1 and CB2 receptor expression can be altered in cancers, often upregulated (for example in hepatocellular carcinoma) and can be correlated with cancer cell invasion, proliferation and apoptosis [3, 4]. However, the roles of cannabinoids and cannabinoid receptor regulation in cancers is not yet fully understood. In particular, cannabidiol (CBD) and Δ⁹-tetrahydrocannabinol (THC) are often studied together [1, 2, 5]. These cannabinoids are usually assessed in combination at a ratio of 1:1 CBD:THC (such as in Sativex®), and sometimes in combination with other anti-cancer agents, such as temozolomide (TMZ). Indeed, phase I/II clinical trials in glioblastoma multiforme (GBM) patients have found that Sativex® was safe to administer with TMZ [6–8], and further studies are underway to study the efficacy of this drug combination with radiotherapy [9, 10]. Cannabinoids are reported to exhibit effects against several cancers. CBD itself has demonstrated activity against colorectal, breast, glioma, cervical and lung cancers [3, 11].

There are varied reports on the anti-cancer mechanisms of action of CBD [5, 11–13]. Whilst CBD is understood to have multiple targets, with a rich and diverse pharmacology, most of the pathways involved are only hypothesised. The suspected pathways involved are *via* transient receptor potential cation channel subfamily V member 2 (TRPV-2), increased reactive oxygen species generation and increased endoplasmic reticulum stress. Some effects have been shown to be reversed following inhibition of CB1 and CB2 receptors, demonstrating some anti-cancer activity of CBD *via* interaction with the endocannabinoid system [1, 3, 5, 11–13]. Additionally, in in vivo mice studies (hippocampus analysis and forced swim tests) the effects of CBD have been reported to involve deoxyribonucleic acid (DNA)-methylation, predominantly at the *C*^5^-cytosine in cytosine-phosphate-guanine (CpG) islands [14, 15]. DNA-methylation has not been reported as a mechanism of anti-cancer activity of CBD, as far as we are aware, and is therefore a hypothesised mechanism of anti-cancer activity. However, the methylation of cytosine in CpG islands indicates that nucleotide base methylation does occur as a result of exposure to CBD, and therefore, DNA-methylation may be a possible mechanism of CBD anti-cancer activity [14, 15]. Inhibition of CB1, CB2 and TRPV-2 receptors has also been shown to reverse some of the anti-cancer effects of CBD, however the pathways involved are not yet fully understood [3, 11].

4’-Fluoro-cannabidiol (4’-F-CBD), also referred to as HUF-101 and PECS-101 in the literature, is a recently synthesised CBD derivative [16, 17]. 4’-F-CBD is reported to exhibit increased potency over CBD in in vivo behavioural assays [16, 18, 19]. Additionally, there is a recent report that 4’-F-CBD can prevent chemotherapy-induced pain [17]. However, to the best of our knowledge, the anti-cancer properties of 4’-F-CBD have not yet been studied.

Glioblastoma multiforme (GBM) is an aggressive grade IV astrocytoma with a dismal prognosis of 5% 5-year survival [20]. Contributing to the poor prognosis is the common resistance of GBM to the standard of care chemotherapy, TMZ. TMZ is a DNA-alkylating agent, predominantly methylating DNA purines at *N*^3^-adenine, *N*^7^- and *O*^6^-guanine positions. *N*-methylation is generally repaired quickly by base excision repair, but *O*-methylation is not [21, 22]. *O*-methylation leads to a mis-pair of guanine with thymine (rather than cytosine) during DNA replication, triggering DNA mismatch repair (MMR), leading to cell death *via* apoptosis or autophagy [23]. There are two major resistance mechanisms to TMZ demonstrated in GBM. Firstly, an over-expression of *O*^6^-methylguanine-DNA methyltransferase (MGMT) allows the cells to repair DNA-methylation at the *O*^6^-guanine position, restoring guanine. Secondly, MMR deficiency allows *O*^6^-methylguanine to be tolerated [22, 24]. One method to try to overcome these common resistance mechanisms to TMZ is to synthesise analogues of the molecule. T25 is a N3-propargyl, C8-thiazole analogue of TMZ, created to overcome resistance by MGMT over-expression. DNA-alkylation with the propargyl group (rather than methyl of TMZ), means that MGMT is not able to recognise and remove the DNA-alkylation, and the cells are therefore still sensitive to treatment [23, 25, 26]. C8-thiazole, replacing carboxamide, has been shown in vitro to enhance drug metabolism and pharmacokinetic (DMPK) properties, including stability; crucially, T25 is not a substrate for P-glycoprotein, an important efflux pump expressed by blood brain barrier (BBB) epithelia [27].

GBM is difficult to treat due to the location, as the physical BBB protects the brain, restricting the movement of most therapeutic agents into the brain [24]. CBD is known to cross the BBB, and many of the observed effects of CBD are a result of interaction with the endocannabinoid system in the brain [28–32]. There are few reports of CBD activity alone against GBM, although these demonstrate a good response, with the concentration required to inhibit cell growth by 50% (GI_50_) ranging from 10.67 ± 0.58 µM against GL216 [33] and 12.75 ± 9.7 µM against U87MG [34–38] to 21.6 ± 3.5 µM against U373MG [36]. More reports investigate the anti-cancer activity of CBD against GBM in combination with THC or TMZ [3, 4, 11, 34, 39]. The combination of CBD and TMZ has been reported to cause both an additive and synergistic response in vitro [35, 40].

However, the few reports of CBD activity alone against GBM demonstrate a good response, with the concentration required to inhibit cell growth by 50% (GI_50_) ranging from 10.67 ± 0.58 µM against GL216 [33] and 12.75 ± 9.7 µM against U87MG [34–38] to 21.6 ± 3.5 µM against U373MG [36].

Using an in vivo U87MG GBM mouse model, when CBD, THC and TMZ were administered in combination, tumour growth was reduced by a larger extent than after administration of TMZ alone [39]. CBD has also been shown to be effective in in vivo GBM models U87, U251, GSC3832 and GSC387 at 15–20 mg/Kg, in combination treatments with THC and TMZ [3, 36, 39, 41–43]. This has been demonstrated after intravenous, intraperitoneal, subcutaneous and oral administration [2, 36]. CBD has also been investigated in combination with radiotherapy in a mouse GL261 model, resulting in significant growth delay (5.5 ± 2.2 mm^3^ at day 21, compared to 48.7 ± 24.9 mm^3^ in the control group) and almost 90% apoptosis [2, 33].

To the best of our knowledge, there are no reports investigating the activity of CBD alone against TMZ-resistant GBM models. However, there is a report of CBD activity against the colorectal cancer cell line, HCT116 [12]. HCT116 cells exhibit a deficiency of MMR and are therefore commonly used as a model to represent this resistance mechanism to (or tolerance to treatment by) TMZ. In the study, CBD was administered alone and found to inhibit cell growth with a GI_50_ of 10.8 µM after 24 h exposure [12]. The common resistance mechanisms to GBM treatment with TMZ prevent the conversion of DNA-methylation to cell death [22, 24]. As discussed, CBD is thought to act *via* multiple pathways [1, 3, 5, 11–13], and therefore may be able to overcome the two major resistance mechanisms to GBM treatment, MGMT over-expression and MMR deficiency.

The aims of this work were to assess the anti-cancer activity of CBD and 4’-F-CBD against GBM. Cells sensitive to TMZ treatment and those representing the two major resistance mechanisms (over-expression of MGMT and MMR deficiency) have been studied to understand whether the cannabinoids‘ activity is impacted by these resistance mechanisms. As a synergistic response of CBD treatment with TMZ has been reported previously, and clinical evaluation of TMZ in combination with Sativex is underway, herein, combination treatments of cannabinoids (CBD and 4’-F-CBD) and TMZ or derivative, T25, were studied. Finally, 3D Orbitrap secondary ion mass spectrometry (3D OrbiSIMS) analysis was used as a novel approach to study the mechanisms of anti-cancer action of CBD. The 3D OrbiSIMS allows label-free imaging at the subcellular level by combining time of flight and Orbitrap detectors for analysis with high spatial resolution and mass resolving power (240,000 at m/z 200) to both analyse the chemistries and visualise their distribution in a sample [44].

## Methods

### Materials

Plant-derived and synthetic CBD were purchased from THC Pharm (Frankfurt, Germany). 1-Fluoropyridinium triflate was purchased from Fluorochem (Derbyshire, UK). Isolute HM-N was purchased from Biogate (Hengoed, UK). Cell lines U373-V and U373-M were supplied by Schering Plough (NJ, USA). Cell lines HCT116 and MRC-5 were purchased from ATCC (VA, USA). RPMI-1640, minimum essential medium, foetal bovine serum (FBS), non-essential amino acids, geneticin G418, gentamicin, L-glutamine, penicillin/streptomycin, sterile Hepes buffer, sterile cell culture sodium bicarbonate, ethylenediaminetetraacetic acid, 10× trypsin- ethylenediaminetetraacetic acid solution, TMZ, ammonium formate, indium tin oxide-coated glass slides, dry dichloromethane, deuterated chloroform (CDCl_3_) and sterile dimethyl sulfoxide were purchased from Sigma Aldrich (Dorset, UK). 3-(4,5-Dimethylthiazol-2-yl)-2,5-diphenyltetrazolium bromide (MTT) was purchased from Alfa Aesar (Heysham, UK). T25 was synthesised within the University of Nottingham by Helen Summers [27]. All other solvents and reagents used were of high performance liquid chromatography grade or higher, purchased from ThermoFisher Scientific (Leicestershire, UK).

### General chemistry

A Buchi Rotavapor consisting of a V-850 vacuum controller, R-210 rotavapor and B-491 heating bath was used for drying. A Biotage SP4 flash chromatography system was used for separation with a normal phase puriFlash (PF-15SIHP-F0004, Interchim, Montluçon, France) column cartridge. A flow rate of 5 mL/min was used with line A (hexane) and line B (20% ether in hexane). The column cartridge was equilibrated with 5% line B for 3 column volumes (CV) first. After equilibration, the product was loaded onto the column. The gradient used was 0–2 CV 5% line B, 2–12 CV 5–10% line B, 12–22 CV10% line B, 22–32 CV 10–20% line B, 32–35 CV 20% line B. Separation was confirmed with thin layer chromatography on silica precoated aluminium backed 60 F_254_ plates (Merck, Darmstadt, Germany) using 6% ether in hexane. Compounds were visualised by a UV lamp at 254 nm.

Liquid chromatography mass spectrometry (LC-MS) was used to verify the product. A Shimadzu UFLCXR system was used with an Applied Biosystems API3000 to visualise spectra. Separation was achieved using a Phenomenex Gemini-NX C18 110 A column (50 mm × 2 mm × 3 µm) at 40 °C. A flow rate of 0.5 mL/min was used with 0.1% formic acid in water in line A and 0.1% formic acid in acetonitrile in line B. The gradient used was 0.0–1.0 min 5% line B, 1.0–3.0 min 5–98% line B, 3.0–5.0 min 98% line B, 5.0–5.5 min 98–5% line B, 5.5–6.5 min 5% line B.

Bruker 400 Ultrashield nuclear magnetic resonance (NMR) was used to assess the product by hydrogen (^1^H) NMR at 400 MHz using CDCl_3_ (δ = 7.26). MestReNova software version 14.2.2 (Mestrelab Research, Santiago de Compostela, Spain) was used to process the data. Chemical shifts (δ) are reported in parts per million (ppm). Coupling constants (*J*) are recorded in Hz, and the multiplicities are described as singlet (s), doublet (d), triplet (t), multiplet (m) or broad (br).

### 4’-Fluoro-cannabidiol

**Fig. 1:**
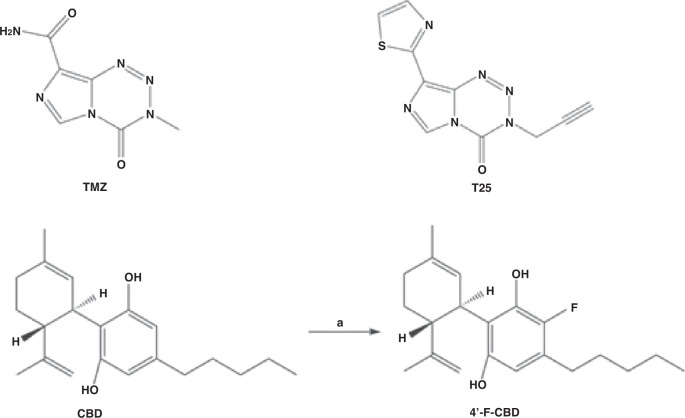
Chemical structures of TMZ, T25, CBD and 4’-F-CBD, and synthesis of 4’-F-CBD.

The synthesis of 4’-F-CBD is shown in Fig. 1 and was first reported by Breuer et al. [16], this method was followed, with modifications to improve the separation of the product from any unreacted CBD.

Synthetic CBD was used as an initial starting point for the synthesis. 1-Fluoropyridinium triflate (79 mg, 0.3 mmol), CBD (100 mg, 0.3 mmol) and 4.5 mL dry dichloromethane were stirred overnight in a nitrogen environment at room temperature. The yellow product was washed with (3 × 5 mL) aqueous sodium bicarbonate (NaHCO_3_). The organic layer was then dried over sodium sulphate (Na_2_SO_4_) anhydrous, filtered and dried onto isolute (1–2 spatulas). Separation of 4’-F-CBD from any unreacted CBD was performed by Biotage SP4 flash chromatography and confirmed by thin layer chromatography.

Characterisation reported by Breuer et al. [16]: total yield (27%), ^1^H NMR (300 MHz, CDCl_3_) δ = 6.17 (s, 1H, Ar), 5.52 (s, 1H), 4.56 (s, 1H), 4.44 (s, 1H), 3.92 (s, 1H), 2.50 (br, 2H), 2.19–2.05 (br, 2H), 1.77 (s, 3H), 0.86 (t, 3H), LC-MS [M + H]^+^ m/z = 332.

Characterisation found: total yield (42%), this is higher than reported due to improved separation by flash chromatography. ^1^H NMR (400 MHz, CDCl_3_) δ = 6.20 (d, *J* = 6.3, 1H, Ar), 5.72 (br, s, 1H, O**H**), 5.56 (d, *J* = 2.6, 1H, C**H** = C), 5.03 (br, s, 1H, O**H**), 4.60 (s, 1H, C**H** = C), 4.47 (s, 1H, C**H** = C), 3.94 (d, *J* = 10.1, 1H, Ar-C**H**), 2.69–2.40 (m, 3H, C**H**_**3**_-C = C), 2.28–2.20 (br, m, 1H, C**H**-C = C), 2.17–2.07 (m, 1H, C**H**-C = C), 1.88–1.75 (m, 2H, C**H**_**2**_), 1.71 (d, *J* = 1.3, 3H, C**H**_**2**_-C**H**), 1.63–1.54 (m, 5H, C**H**_**3**_, C**H**_**2**_), 1.35 (dd, *J* = 7.3, 2.0, 2H, C**H**_**2**_), 1.35–1.23 (m, 2H, C**H**_**2**_), 0.91 (t, *J* = 6.8, 3H, C**H**_**3**_). Whilst Breuer et al. [16] did not report all ^1^H NMR peaks, those they did report match those found, and the additional peaks could all be assigned to the structure as described. LC-MS: [M + H]^+^ calculated m/z = 332.5, found m/z = 332.9, retention time: 3.26 mins, purity 95%. LC-MS characterisation matches that reported by Breuer et al. [16].

### Cell culture

Human GBM cell lines U373-V (MGMT-low, +MMR) and U373-M (+MGMT, +MMR) and human colorectal cancer cell line HCT116 (MGMT-low, −MMR) were used in this work. Cell lines U373-V and U373-M were cultured in RPMI-1640 medium supplemented with 10% FBS, 1% non-essential amino acids, 50 µg/mL gentamycin and 400 µg/mL G418. Cell line HCT116 was cultured in RPMI-1640 medium supplemented with 10% FBS and 1% penicillin/streptomycin. Non-tumourigenic foetal lung fibroblasts (MRC-5) were cultured in minimum essential medium supplemented with 10% FBS, 1% non-essential amino acids, 1% penicillin/streptomycin, 2 mM L-glutamine, 10 mM Hepes buffer and 0.075% sodium bicarbonate. All cell lines were cultured in an incubator with 5% CO_2_ at 37 °C.

### MTT assay

The MTT assay was used to evaluate the growth and viability of all cell lines used upon treatment with CBD and 4’-F-CBD alone and combinations of CBD and TMZ, CBD and T25, 4’-F-CBD and TMZ, and 4’-F-CBD and T25. Briefly, cells were seeded into 96-well plates at the following densities: 3 days‘ exposure: all cell lines: 3 × 10^3^ cells/well; 6 days‘ exposure: U373-V and U373-M cells: 650 cells/well, HCT116 and MRC-5 cells: 400 cells/well. After the cells were allowed to attach overnight, they were exposed to test agents for either 3 or 6 days. MTT assays were performed at the time of treatment (T0) and following the exposure time for cells treated and non-treated controls. MTT was added, and following 2 h incubation, the formazan crystals were dissolved in 150 µL sterile dimethyl sulfoxide and absorbance was read at λ = 570 nm on a PerkinElmer EnVision plate reader. GI_50_ and combination index (CI) values were calculated using Equations (1)–(3) outlined in Supplementary information 1.

### Cell viability

Results of the MTT assays were confirmed by viable cell count assays. Cells were seeded into 6-well plates at the following densities: U373-V and U373-M cells: 4 × 10^4^ cells/well, HCT116 and MRC-5 cells: 2 × 10^4^ cells/well. After the cells were allowed to attach overnight, they were exposed to test agents for either 3 or 6 days. Following the exposure time, cells were washed with PBS and harvested with trypsin-ethylenediaminetetraacetic acid solution. The viable cells were then counted using a haemocytometer under a Nikon Eclipse TS100 microscope.

### Preparation of cells for 3D OrbiSIMS analysis

Cell samples were prepared for analysis by 3D OrbiSIMS following a method based on Newman et al. [45]. U373-V cells treated with CBD, CBD and TMZ, and CBD and T25 were assessed by 3D OrbiSIMS.

Indium tin oxide-coated glass slides were placed into a petri dish and seeding U373-V cells at a density of 1.6 × 10^5^ cells/well into the dish. Petri dishes were placed in the incubator at 5% CO_2_, 37 °C. Cells were exposed to the GI_50_ value of test agents for 3, 6, 24 and 72 h to be able to compare to the MTT assays. For cells treated with a combination of test agents, the concentrations were based on combination MTT assays to represent ~75% growth inhibition, shown in Table 1.

**Table 1:** Concentration of test agents for preparation of cell exposure, dosed in combination for 3D OrbiSIMS analysis.

Test agent A	Test agent A concentration (µM)	Test agent B	Test agent B concentration (µM)
CBD	11	**TMZ**	2
CBD	7	**T25**	14

Following the exposure time, the slides were harvested. The cells were washed (3× 1 mL) with 150 mM ammonium formate solution at pH 7.4. The glass slides were then dipped into liquid nitrogen and freeze-dried in a benchtop freeze dryer (VirTis SP Scientific Sentry 2.0) at −50 °C for 1 h. Once removed from the freeze drier, the slides were sealed in petri dishes with parafilm and stored at −80 °C until analysis.

### 3D OrbiSIMS analysis

The 3D OrbiSIMS technique uses a HybridSIMS instrument (IONTOF GmbH), which incorporates both time of flight and Q Exactive HF Orbitrap analysers. Samples were analysed using the single ion beam Orbitrap depth profiling mode, utilising a 20 keV Ar_3000_^+^ gas cluster ion beam of 20 µm diameter (duty cycle of 4%) and a target current of 0.2 nA. Both positive and negative mode ion polarity spectra were acquired with a mass range of m/z = 75–1125. The profile was performed over an area of 200 × 200 μm using random raster mode. The injection time was set to 500 ms and 80 scans were taken for each analysis over an average of 120 s. A low energy electron floodgun was used for charge compensation, additionally, the pressure in the main chamber was regulated using Ar gas to 9 × 10^−7^ mbar to enhance the charge compensation. The mass resolution was 240,000 at m/z 200.

3D OrbiSIMS data were acquired and analysed using SurfaceLab 7 software (IONTOF GmbH, Münster, Germany). Peak lists were automatically generated for all of the spectra with a minimum count value applied of 10,000 and subsequently combined using the ‘union’ function with a catch mass radius of 2 ppm. All data were normalised to the total ion count (TIC) of that analysis. All assignments are based on accurate mass to within 2 ppm, and those reported throughout are putative. Data were chemically filtered using molecular formula prediction software, SIMS-MFP version 1.1 (University of Nottingham, Nottingham, UK) [44], into groups containing fatty acids (C_n_H_n_O_2_), sulfatides (C_n_H_n_N_1_O_11-12_S_1_) and glycerophospholipids (C_n_H_n_O_8/13_P or C_n_H_n_NO_7-10_P) [46]. Data groups were then analysed using multivariate analysis software, simsMVA [47]. The data groups were mean-centred, and the principal component analysis (PCA) function was used in algorithm mode, retaining all components. The scores and variance were used to find principal components exhibiting differences between the groups, and loadings allowed visualisation of the principal components.

### Statistical analysis

Chemical structures and schemes were prepared using ChemDraw version 21.0.0 (PerkinElmer Informatics, MA, U.S.A.). One-way ANOVA with Dunnett’s multiple comparisons, or multiple t-tests where appropriate were performed in Prism version 9.3.1 (GraphPad, CA, U.S.A.) to assess the significant differences between sample groups. Differences were considered statistically significant when the *p*-value was <0.05 (α = 0.05). All data (*n* ≥ 3 independent experimental repeats; *n* = 5 internal sample replicates) are represented as mean ± standard deviation (SD).

## Results

### Cancer cell growth inhibition by cannabinoids

**Fig. 2:**
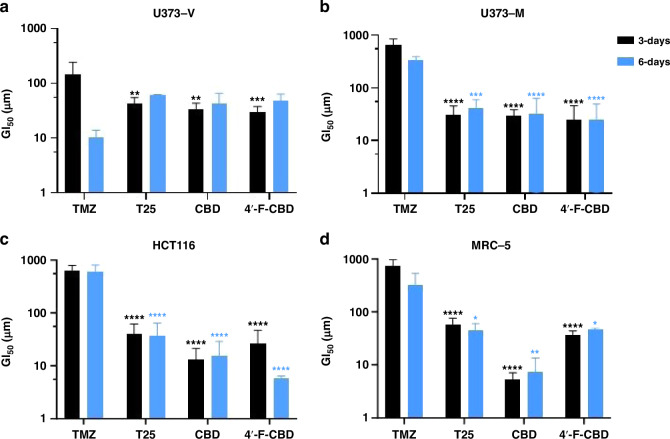
GI_50_ values of cannabinoids CBD and 4’-F-CBD compared to DNA-alkylating agents TMZ and T25 against (**A**) U373-V (GBM control, −MGMT, +MMR, TMZ sensitive), (**B**) U373-M (GBM, +MGMT, +MMR, TMZ resistant), (**C**) HCT116 (-MGMT, -MMR, TMZ resistant) and (**D**) MRC-5 (non-tumourigenic) after 3- and 6-days exposure. Data measured by MTT assay and confirmed by cell count assay. Data are presented as mean ± SD, three independent repeats of *n* = 5. One-way ANOVA was performed, comparing test agents to TMZ, α = 0.05, **p < 0.01, ***p < 0.001, ****p < 0.0001. Differences in GI_50_ compared to TMZ are shown for for both 3- and 6- days exposure.

The anti-cancer activity of cannabinoids CBD and 4’-F-CBD was assessed against a vector control GBM cell line (U373-V) and two cell lines representing common resistance mechanisms to treatment with TMZ (U373-M, MGMT-transfected U373-V isogenic partner, and MMR-deficient HCT116 colorectal cancer). Exposure periods of 6-days as well as 3-days were studied because TMZ is understood to require at least one cell cycle in order to exhibit its cytotoxic effect [22]. This is observed in Fig. 2, where the GI_50_ of TMZ against the U373-V cell line falls significantly (*p* < 0.001) from 147 ± 55 µM after 3-days exposure to 10 ± 2 µM following 6-days exposure. After 3-days exposure, T25, CBD and 4’-F-CBD exhibited significantly lower GI_50_ values compared to TMZ against all cell lines studied. For U373-M and HCT116 cell lines (demonstrating resistance to TMZ treatment), both cannabinoids and T25 also showed significantly lower GI_50_ values than TMZ following 6-days exposure. T25 data corroborate results first reporting T25 potency in cell lines demonstrating clinical mechanisms of resistance to TMZ [27] and are consistent with the hypothesis that propargyl lesions are neither removed (by MGMT) nor tolerated in MMR-deficient cells [48].

To obtain preliminary indications of cancer-selectivity, test agents were also assessed against non-tumourigenic MRC-5 fibroblasts, as shown in Fig. 2. TMZ was shown to be the least active, with a GI_50_ of 323 µM after 3-days exposure, or 724 µM after 6-days exposure. CBD appears to be the most potent, with a GI_50_ of 5 µM and 7 µM (3- and 6-days exposure). 4’-F-CBD and T25 both demonstrated GI_50_ values between 37 and 58 µM.

### Synergy of cannabidinoids with imidazotetrazine anti-cancer agents

**Fig. 3:**
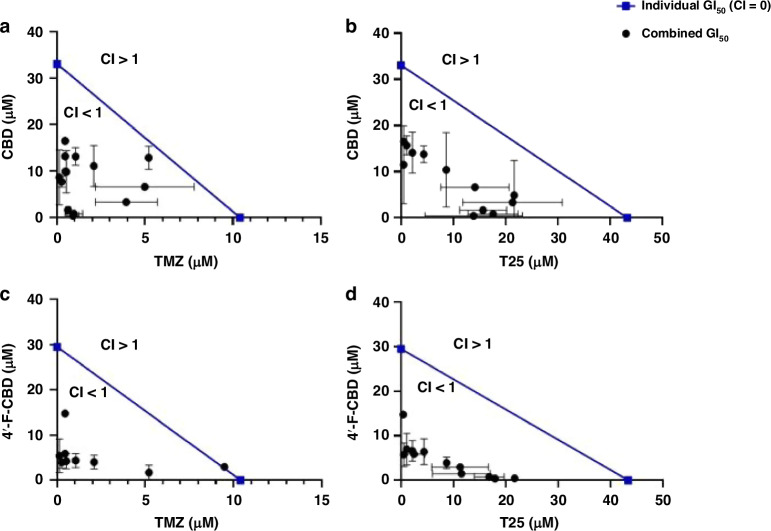
Isobolograms representing the combined effect of (**A**), CBD and TMZ, (**B**) CBD and T25, (**C**) 4’-F-CBD and TMZ, and (**D**) 4’-F-CBD and T25 against U373-V (GBM control, −MGMT, +MMR) after 3-days exposure. Data measured by MTT assays and confirmed by cell count assays. Data presented as mean ± SD, three independent repeats of *n* = 5.

Combination treatments of CBD with TMZ or T25 against the three cell lines were studied by MTT assays and confirmed by cell count assays. The combination indices (CIs) indicating the cell response to the combined treatments are shown in Table 2. Briefly, CI = 1 indicates an additive response, CI > 1 is antagonistic and CI < 1 shows a synergistic response. The data in Table 2 are demonstrated as a graphical example in Fig. 3, where the isobolograms of combinations against the U373-V cell line are shown.

**Table 2:** CIs of cannabinoids (CBD or 4’-F-CBD) administered in combination with TMZ or T25 after 3- or 6-days exposure against U373-V (GBM control, −MGMT, +MMR, TMZ sensitive), U373-M (GBM, +MGMT, +MMR, TMZ resistant) and HCT116 (−MGMT, −MMR, TMZ resistant) cell lines.

Combination	Treatment time (days)	Combination Index (CI)		
		U373-V	U373-M	HCT116
		−MGMT, +MMR	+MGMT, +MMR	−MGMT, −MMR
CBD/TMZ	3	0.10–0.89	0.23–0.74	0.11–1.06
	6	0.12–0.76	0.21–0.74	0.05–0.95
CBD/T25	3	0.33–0.65	0.22–0.57	0.53–0.93
4’-F-CBD/TMZ	3	0.16–1.01	0.13–0.59	0.37–0.74
	6	0.09–0.53	0.05–0.50	0.48–0.79
4’-F-CBD/T25	3	0.21–0.52	0.19–0.75	0.37–0.79

Data represented as a range for the different combination concentrations tested. The full CI data for each combination are shown in Supplementary Information 2.

Remarkable synergistic responses were encountered in all 3 cell lines when CBD and an imidazotetrazine agent (TMZ or T25) were combined. Table 2 shows that only against the HCT116 cell line was there a combination that did not provide a synergistic response, CI = 1, when TMZ (304.5 µM) was used with CBD (7.5 µM) after only 3-days exposure (when TMZ is less effective, as shown in Fig. 2). However, when HCT116 cells were treated with test agents for 6-days (required to observe the full effects of TMZ in TMZ-sensitive cells), the lowest CI (greatest synergy) was observed following exposure to CBD (1.3 µM) and TMZ (0.5 µM). Table 2 and Fig. 3 also show that the combination of CBD and T25 provided the most consistent response, with a smaller range in CI values (e.g. MGMT + U373-M 0.22 ≤ CI ≤ 0.57).

Combination treatments of 4’-F-CBD with TMZ or T25 were also assessed against the three cell lines, showing that the combination of 4’-F-CBD with TMZ or T25 resulted in a synergistic response in all three cell lines. The only exception was following 3-days exposure of U373V cells to 4’-F-CBD and TMZ. Similarly to the only additive response observed in the CBD combination studies, this was at low concentrations of the test agents, and at 3-days where TMZ has not yet been able to exhibit its full effect. Indeed, following 6 days‘ exposure to 4’-F-CBD and TMZ, the lowest CI of 0.09 was observed in U373-V cells (Table 2). Multiple mechanisms which may contribute to such synergy are considered in the discussion.

### Indications of anti-cancer mechanisms of cannabidiol activity by 3D OrbiSIMS

**Fig. 4:**
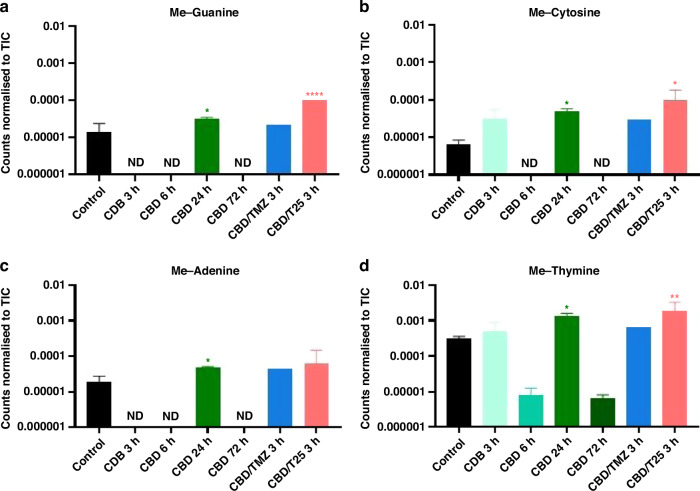
3D OrbiSIMS analysis of U373-V cells exposed to CBD for 3, 6, 24 and 72 h, CBD and TMZ for 3 h, CBD and T25 for 3 h and a non-treated control. Data presented as peak intensity (secondary ion counts) normalised to the TIC for (**A**) methyl-guanine (C_6_H_6_N_5_O^−^), (**B**) methyl-cytosine (C_5_H_6_N_3_O^−^), (**C**) methyl-adenine (C_6_H_6_N_5_^−^) and (**D**) methyl-thymine (C_5_H_5_N_2_O_2_^−^). Data presented as an average of *n* = 3 technical repeats. ND not detected. One-way ANOVA was performed, α = 0.05, *p < 0.05, **p < 0.01, ****p < 0.0001 to compare treated samples to the control. The peak intensities and deviation of peak assignment is shown in Supplementary Information 3.

U373-V cells exposed to CBD were investigated by the 3D OrbiSIMS technique with cells analysed following exposure to CBD either alone, with TMZ or with T25 for up to 3-days. This technique was not used to measure cytotoxicity, but to shed light on potential anti-cancer mechanism of action of CBD. Using the spectra acquired, a targeted search for secondary ions indicative of the suspected mechanisms of action was conducted including glutathione (C_10_H_16_N_3_O_6_S^-^) as an indicator of oxidative stress [49], ceramide (C_63_H_124_NO_6_S^-^) as an indicator of CB1 activity [50], and anandamide (C_22_H_36_NO_2_^-^) as an indicator of interaction with the endocannabinoid system [51]. These were not observed with 3D OrbiSIMS analysis; however, DNA and methylated-DNA ions were observed. From the secondary ion intensity values shown in Fig. 4, it can be seen that cells exposed to CBD for 24 h exhibited significantly higher methylated-DNA content compared to the control samples of non-treated cells. Figure 4 also shows that following 3 h exposure of the cells to CBD with T25, methylated-guanine, cytosine and thymine were also observed at significantly higher levels than in the control sample. T25 is thought to create propargyl-adducts on DNA, not methyl lesions. Table 3 demonstrates this for the first time, showing secondary ions related to propargylated-DNA were found following exposure of U373-V cells to CBD and T25. Significant differences were not observed following exposure to CBD alone for 3, 6 or 72 h. Supplementary Information 3 shows more details of the detection of methylated-DNA shown in Fig. 4.

**Table 3:** 3D OrbiSIMS analysis of U373-V cells exposed to CBD for 3 h, CBD and T25 for 3 h and a non-treated control.

Sample		Propargyl-guanine	Propargyl-cytosine	Propargyl-adenine	Propargyl-thymine
	Formula	C_8_H_6_N_5_O^−^	C_7_H_6_N_3_O^−^	C_8_H_6_N_5_^−^	C_7_H_5_N_2_O_2_^−^
	m/z	188.1675	148.1431	172.1685	149.1276
Control		0	1.09 × 10^−5^ ± 2.70 × 10^−6^	0	7.56 × 10^−6^ ± 2.65 × 10^−6^
		–	0.4	–	−0.3
CBD 3 h		0	0	0	0
		–	–	–	–
CBD/T25 3 h		4.16 × 10^−5^ ± 0	1.77 × 10^−4^ ± 1.47 × 10^−4^	3.48 × 10^−5^ ± 0	1.16 × 10^−4^ ± 9.82 × 10^−5^
		−0.1	0.1	0.1	−0.4

Data presented as an average of *n* = 3 technical repeats. One-way ANOVA was performed, α = 0.05, to compare treated samples to the control, no significant differences were found.

Peak intensity (secondary ion counts) normalised to the TIC, with deviation below in ppm, of propargylated-DNA.

Further analysis of the 3D OrbiSIMS data using PCA revealed that cells exposed to CBD alone exhibited an increase in fatty acid content. Following exposure for 3 and 6 h, an increase in palmitic, stearic and octatriacontanoic acids was observed, as well as a decrease in oleic acid. After 72 h exposure, only an increase in palmitic acid was observed. The cells exposed to CBD and T25 also exhibited a change in the fatty acid composition, showing an increase in palmitic and octatriacontanoic acids, and exposure of cells to CBD and TMZ resulted in an increase in arachidonic, cinnamic and palmitic acids. A detailed illustration of the PCA conducted using the 3D OrbiSIMS data demonstrating the difference in fatty acid composition of samples is shown in Supplementary Information 4.

Potential implications of changes in fatty acid composition are discussed.

## Discussion

Exploration of the anti-cancer effects of cannabinoids is a growing area of research. CBD has been shown to exhibit anti-tumour properties including against breast, colorectal, lung carcinomas and GBM [5, 12]. The ability of CBD to inhibit GBM cell growth in vitro is usually studied in combination with either THC or TMZ [34, 39]. This has led to phase I/II clinical trials in GBM patients [6–8]. Further clinical trials are underway to study the efficacy of combinations of radiotherapy, chemotherapy with TMZ and a mixture of CBD and THC, against GBM [9], as well as daily administration of CBD with TMZ [10]. As discussed, the anti-cancer activity of CBD alone against GBM has been studied in cell lines including U87MG (GI_50_ = 12.75 ± 9.7 µM) [34–38], GL216 (GI_50_ = 10.67 ± 0.58 µM) [33] and U373MG (GI_50_ = 21.6 ± 3.5 µM) [36]. These cell lines do not possess MGMT over-expression or MMR deficiency that comprise major GBM resistance mechanisms to TMZ, represented in this work by human GBM U373-M and colorectal cancer HCT116 cell lines, respectively. CBD’s anti-cancer activity has been studied against the HCT116 cell line previously for its effects against colorectal cancer [12]. In this work, HCT116 cells were utilised to represent the second major resistance mechanism to TMZ, a deficiency in MMR. To the best of our knowledge, the anti-cancer properties of 4’-F-CBD have not been studied before. The potential advantages of treating GBM with 4’-F-CBD, compared to CBD, are, briefly, that 4’-F-CBD is reported to have increased potency in in vivo behavioural assays compared to CBD, suggesting potentially increased binding at the molecular level, or increased delivery to the brain [16, 18, 19]. The fluorine atom on 4’-F-CBD also offers imaging and theranostic potential [52, 53].

The activity of all agents was assessed against non-tumourigenic MRC-5 fibroblasts to indicate putative cancer-selectivity and therapeutic window. Figure 2 demonstrates that TMZ showed the greatest, and CBD the least cancer-selectivity (GI_50_ values = 724 µM and 7 µM, respectively, following 6-days exposure). Therefore, although CBD is known to be safe for humans (≥6000 mg/Kg with no adverse side effects [54]), for cancer treatment, a more cancer-selective drug delivery system may be considered [55]. Against the U373-V cell line, the TMZ GI_50_ falls from 147 ± 55 µM after 3-days to 10 ± 2 µM after 6-days exposure (Fig. 2). This is consistent with TMZ’s understood mechanism [56] as TMZ must undergo ring opening to MTIC, before it is able to methylate DNA, most impactfully at *O*^6^-guanine [21, 22]. *O*^6^-Methylation leads to a guanine-thymine (rather than cytosine) mis-pair during DNA replication, triggering MMR and ultimately leading to cell death *via* apoptosis or autophagy [23]. This process comprises multiple rounds of futile DNA incision and thymine re-insertion before DNA-replication fork collapse, thus 6-days‘ exposure is required to realise the impact of TMZ treatment. For the 2 cell lines representing common (clinical) resistance mechanisms (U373-M and HCT 116), TMZ GI_50_ > 300 µM were as expected and demonstrated in the literature [22].

Interestingly, for imidazotetrazine analogue T25, CBD and 4’-F-CBD growth inhibitory effects after 3-days exposure against all 3 cancer cell lines were observed. GI_50_ values < 50 µM for CBD, 4’-F-CBD and T25 were consistent across cancer cell lines studied, and all values were significantly (*p* < 0.001) lower than that of TMZ against the two cell lines displaying TMZ resistance. This has been demonstrated previously within our group for T25 [27], as the molecule was designed to overcome resistance mechanisms associated with TMZ treatment, creating larger propargyl DNA adducts that escape MGMT-mediated removal and tolerance following MMR-loss. The activity of CBD alone against HCT116 has also been reported in the literature, supporting the thesis that CBD activity is not impacted by resistance to TMZ conferred by MMR deficiency [12]. However, this is the first time that cannabinoids have been shown to overcome the often-seen inherent- (and occasionally acquired- [57]) resistance to TMZ conferred by MGMT. Additionally, 4’-F-CBD demonstrated increased cancer-selectivity compared to CBD (Fig. 2) and may ultimately provide a safer treatment option. These are encouraging data, as the poor prognoses for GBM patients demonstrate the need for new treatments.

As discussed, synergy has previously been demonstrated between CBD and TMZ against GBM cell lines U87MG and U251 [35, 40, 58]. However, Deng et al. reported that only certain concentrations resulted in a synergistic combination, whilst others resulted in an additive response [35]. The work reported herein confirms synergy in the U373-V (TMZ-sensitive) cell line, and in the two cell lines harbouring clinical resistance mechanisms to TMZ for the first time. The CBD/TMZ combination demonstrated remarkable synergistic responses with CIs as low as 0.21 and 0.05 in U373-M and HCT116 cell lines, respectively (Table 2). Against MMR-deficient HCT116 cells, at high TMZ concentrations, the combination resulted in an additive response. This analysis indicates that TMZ does not impact growth inhibition, and that CBD is driving the response. This suggests that CBD is the predominant cause of growth inhibition, potentially re-sensitising the cells to TMZ. Mechanisms by which CBD may potentiate sensitivity to TMZ include TRPV2 channel activation by CBD, reduction of extracellular vesicles‘- (EV)-mediated drug expulsion from cells, enhanced DNA-damaging reactive oxygen species‘ (ROS) generation, and down-regulation of RAD51 DNA repair protein, evidenced in the literature [56, 59, 60] but as yet unstudied in the work described herein. Some or all of these mechanisms may result in observed synergy between TMZ and CBD.

T25, able to overcome the two major resistance mechanisms to TMZ, also demonstrates synergy in combination with CBD, eliciting enhanced activity in TMZ resistant models (Fig. 2). The CBD/T25 combination yielded a synergistic response at all concentrations tested for all cell lines, moreover, CIs were lower for this combination than for the CBD/TMZ combination (<0.57 compared to <0.74 in U373-M, respectively). The enhanced synergy in the MGMT positive TMZ-resistant model is likely due to the increased activity of T25 compared to TMZ. This combination has not been studied before, mechanisms need to be resolved, yet the low CIs demonstrate promise for GBM treatment.

The combined treatment of CBD with T25 was investigated by 3D OrbiSIMS analysis. Propargylated-DNA (expected to occur following exposure to T25) was found in samples treated with CBD and T25 (Table 3). In particular, propargyl-guanine and propargyl-adenine were found to be present in treated samples. This provides evidence of the activity of T25, and is consistent with alkylation sites induced by N3-propargyl imidazotetrazine analogue and detected by *Thermo aquaticus* (TAQ) polymerase stop assays on runs of guanine residues [48].

Methylated-DNA was also found to be present (at 24 h following exposure to CBD, and 3 h following exposure to CBD and T25, Fig. 4); methyl-guanine, methyl-cytosine and methyl-thymine were all significantly higher than in the non-treated control sample. As T25 is expected, and shown here, to deposit propargyl groups on DNA, the methylated-DNA could be a result of CBD activity. Of particular interest is methyl-cytosine. Methyl-cytosine at the *C*^5^-position of CpG islands is reported to occur after CBD exposure, however the role of CpG methylation in CBD activity is not yet clear [14, 15]. Additionally, CpG islands are abundant in promoter genes, including the *MGMT* promoter [61]. Methylated *MGMT* promoter is an evidenced indicator of the prognosis of GBM response to therapy [62]. *MGMT* promoter methylation silences the gene, MGMT protein is not expressed, and the tumours are more sensitive to TMZ treatment [63]. The methyl-cytosine evidenced herein by exposure of GBM cells to CBD could potentially occur at CpG islands on *MGMT* promoters. If so, this could effectively silence MGMT, possibly contributing to the synergy observed in exposure of the cells to CBD with TMZ.

The presence of methylated-DNA at high OrbiSIMS ion intensities may represent one mechanism of anti-cancer action of CBD. DNA damage by methylation can result in mismatched pairs during replication and ultimately, lead to cell death [64–66]. To the best of our knowledge, this is the first evidence of methylated-DNA as a potential anti-cancer mechanism of action of CBD. As discussed, MMR deficiency (as in the HCT116 cell line) means that mis-matched pairs are tolerated. Therefore, this work indicates that CBD may re-sensitise MMR-deficient cells to *O*^6^-Me lesions. The synergy between CBD and TMZ or T25 indicates that CBD also acts *via* a pathway other than DNA alkylation (the mechanism of action of imidazotetrazine compounds). The increase in palmitic, arachidonic and cinnamic acids observed in cells exposed to CBD is associated with oxidative stress (ROS generation) and decreased GBM cell viability [57, 66]. This supports the hypothesis that oxidative stress is enhanced in cells treated with CBD/imidazotetrazine combinations. Cells treated with CBD were also found to contain decreased oleic acid compared to the non-treated control. Oleic acid has been shown to increase glucose utilisation and stimulate GBM cell growth [65]. However, oleic acid is thought to increase the permeability of the BBB by interacting with the membranes of brain capillary endothelial cells, which form the BBB, therefore, a reduction in oleic acid would impair BBB permeability [67, 68]. Nevertheless, there are reports that oleic acid decreases P-glycoprotein (P-gp)-mediated drug efflux [69]. Thus, reduced oleic acid could potentiate TMZ (a P-gp substrate) levels in the brain. These findings indicate that the anti-cancer activity of CBD involves a rich and diverse pharmacology, as is suggested in the literature [5, 12, 13].

The mechanism of action of 4’-F-CBD was not investigated, however, as the molecular structures of the cannabinoids are similar (Fig. 1), it would be reasonable to suggest that the activity of 4’-F-CBD could be a result of similar pathway(s‘) activation to those of CBD. Synergy was achieved in all cell lines following exposure to 4’-F-CBD and TMZ after both 3- and 6-days exposure (Table 2). Only the highest concentration of TMZ in (TMZ-sensitive) U373-V cells resulted in an additive response; all other concentrations demonstrated a synergistic response (Fig. 3). Therefore, the 4’-F-CBD/TMZ combination produced increased synergy over CBD/TMZ in all cell lines apart from U373V cells after 3-days treatment. 4’-F-CBD/T25 combinations demonstrated high synergistic responses; CIs are not significantly different from CBD/T25 combinations.

The work reported herein shows the promise cannabinoids offer for GBM treatment. Application of 3D OrbiSIMS demonstrates the potential of this technique to elucidate the mechanism(s) of anti-cancer activity of CBD. Further work is proposed to fully investigate the mechanisms proposed in this work. Taq-polymerase stop assays could be conducted to interrogate the intensity of alkyl-guanine, following treatment with cannabinoids in combination with imidazotetrazines TMZ and T25. Analysis of *O*^*6*^-methylguanine adduct burden in cells would also be useful, where comparisons of cells exposed to TMZ alone or in combination with CBD. Additionally, 3D OrbiSIMS has proved beneficial, the technique is not chemically biased and generates a range of different ions simultaneously, so is a good starting point for complex questions which do not have a known direction for analysis. It is also relatively high throughput for in vitro studies. Following mechanistic studies, understanding in vivo PK and biodistribution of 4’-F-CBD will be necessary before investigating efficacy in in vivo models.

GBM represents an unmet clinical need. Inherent or acquired resistance to standard of care alkylating agent TMZ chemotherapy thwarts successful treatment. This work demonstrates for the first time that CBD and 4‘-F-CBD are able to overcome major resistance mechanisms to TMZ, MGMT over-expression and MMR-deficiency. Moreover, the promising in vitro synergy described between imidazotetrazines (TMZ, T25) and cannabinoids (CBD, 4‘-F-CBD) indicate this approach could improve treatment options for GBM patients.

## Supplementary information

Supplementary information

## Data Availability

The datasets generated and analysed during the current study are available in the supplementary information or available upon request.
