# Supplementary material for: Cannabidiol and fluorinated derivative anti-cancer properties against glioblastoma multiforme cell lines, and synergy with imidazotetrazine agents
Source: BJC Rep. 2024 Sep 9;2:67. doi: 10.1038/s44276-024-00088-0 (PMC11524125; doi:10.1038/s44276-024-00088-0)

**Supplementary information 1**

Equations used to calculate the concentration required to inhibit cell growth by 50% (GI_50_) and combination index (CI) values using data collected in 3-(4,5-Dimethylthiazol-2-yl)-2,5-diphenyltetrazolium bromide (MTT) assays.

$GI50A= \left( \frac{\left( C-T0 \right)}{2} \right)$ + T0 **Equation (1)**

GI_50_ $= \left( \left( \frac{(E-D)}{(X-Y)} \right)*\left( X-GI50A \right) \right)+D$ **Equation (2)**

Where:

GI50A = Absorbance at GI_50_

C = Mean absorbance of control (cells only)

T0 = Mean absorbance of control (T0)

X = High absorbance (absorbance at data point just above GI50A)

Y = Low absorbance (absorbance at data point just below GI50A)

D = High concentration (concentration at absorbance data point just above GI50A)

E = Low concentration (concentration at absorbance data point just below GI50A)

$CI= \left( \frac{D1}{DX1} \right)+ \left( \frac{D2}{DX2} \right)$ **Equation (3)**

Where:

CI = Combination index

D1 = GI_50_ of test agent A when in combination with test agent B

DX1 = GI_50_ of test agent A alone

D2 = GI_50_ of test agent B when in combination with test agent A

DX2 = GI_50_ of test agent B alone

**Supplementary information 2**

Full combination index (CI) data of cannabinoids CBD and 4’-F-CBD administered in combination with TMZ or analogue, T25, after 3- or 6-days exposure against U373-V, U373-M and HCT116 cell lines. The data are presented as mean, 3 independent repeats of n = 5.

| Exposure time (days) | Test Agent A | Test Agent B | Test Agent B Concentration (% GI_50_) | Cell line | | |
| --- | --- | --- | --- | --- | --- | --- |
|  |  |  |  | U373-V | U373-M | HCT116 |
| 3 | TMZ | CBD | 1.25 | 0.10 | - | - |
|  |  |  | 2.5 | 0.12 | - | - |
|  |  |  | 5 | 0.11 | - | - |
|  |  |  | 10 | 0.48 | - | 0.11 |
|  |  |  | 20 | 0.68 | - | 0.22 |
|  |  |  | 30 | 0.34 | 0.35 | - |
|  |  |  | 40 | 0.44 | 0.40 | - |
|  |  |  | 50 | 0.54 | 0.50 | 0.51 |
|  | CBD | TMZ | 1.25 | 0.27 | 0.23 | 0.83 |
|  |  |  | 2.5 | 0.26 | 0.24 | 0.82 |
|  |  |  | 5 | 0.35 | 0.25 | 0.67 |
|  |  |  | 10 | 0.50 | 0.33 | 0.93 |
|  |  |  | 20 | 0.54 | 0.45 | 0.91 |
|  |  |  | 50 | 0.89 | 0.74 | 1.06 |
| 6 | TMZ | CBD | 1.25 | 0.67 | - | - |
|  |  |  | 2.5 | 0.76 | - | - |
|  |  |  | 5 | 0.57 | - | 0.05 (1 repeat) |
|  |  |  | 10 | 0.34 | 0.28 | 0.10 (1 repeat) |
|  |  |  | 20 | 0.29 | 0.28 | 0.22 (2 repeats) |
|  |  |  | 50 | 0.66 | 0.69 | 0.50 (1 repeat) |
|  | CBD | TMZ | 1.25 | 0.12 | 0.22 | 0.45 |
|  |  |  | 2.5 | 0.12 | 0.21 | 0.49 |
|  |  |  | 5 | 0.14 | 0.25 | 0.59 |
|  |  |  | 10 | 0.22 | 0.30 | 0.78 |
|  |  |  | 20 | 0.30 | 0.38 | 0.74 |
|  |  |  | 50 | 0.61 | 0.74 | 0.95 |
| 3 | TMZ | 4’-F-CBD | 1.25 | - | - | - |
|  |  |  | 2.5 | - | - | - |
|  |  |  | 5 | - | - | - |
|  |  |  | 10 | 1.01 | 0.22 | - |
|  |  |  | 20 | 0.24 | 0.20 | - |
|  |  |  | 50 | 0.54 | 0.50 | 0.50 |
|  | 4’-F-CBD | TMZ | 1.25 | 0.20 | 0.13 | 0.37 |
|  |  |  | 2.5 | 0.16 | 0.13 | 0.37 |
|  |  |  | 5 | 0.19 | 0.16 | 0.46 |
|  |  |  | 10 | 0.25 | 0.20 | 0.48 |
|  |  |  | 20 | 0.34 | 0.28 | 0.65 |
|  |  |  | 50 | 0.56 | 0.59 | 0.74 |
| 6 | TMZ | 4’-F-CBD | 1.25 | 0.99 | - | - |
|  |  |  | 2.5 | 0.11 | - | - |
|  |  |  | 5 | 0.09 | 0.12 | - |
|  |  |  | 10 | 0.14 | 0.10 | - |
|  |  |  | 20 | 0.24 | 0.20 | - |
|  |  |  | 50 | 0.54 | 0.50 | 0.50 |
|  | 4’-F-CBD | TMZ | 1.25 | 0.09 | 0.05 | 0.48 |
|  |  |  | 2.5 | 0.11 | 0.06 | 0.51 |
|  |  |  | 5 | 0.12 | 0.08 | 0.51 |
|  |  |  | 10 | 0.18 | 0.15 | 0.56 |
|  |  |  | 20 | 0.28 | 0.24 | 0.67 |
|  |  |  | 50 | 0.53 | 0.53 | 0.79 |
| 3 | T25 | CBD | 1.25 | 0.33 | 0.36 | 0.65 |
|  |  |  | 2.5 | 0.43 | 0.34 | 0.76 |
|  |  |  | 5 | 0.41 | 0.40 | 0.65 |
|  |  |  | 10 | 0.59 | 0.38 | 0.83 |
|  |  |  | 20 | 0.53 | 0.43 | 0.74 |
|  |  |  | 50 | 0.51 | 0.51 | 0.75 |
|  | CBD | T25 | 1.25 | 0.36 | 0.22 | 0.53 |
|  |  |  | 2.5 | 0.50 | 0.31 | 0.54 |
|  |  |  | 5 | 0.48 | 0.35 | 0.57 |
|  |  |  | 10 | 0.52 | 0.38 | 0.77 |
|  |  |  | 20 | 0.51 | 0.46 | 0.68 |
|  |  |  | 50 | 0.65 | 0.57 | 0.93 |
|  | T25 | 4’-F-CBD | 1.25 | 0.43 | 0.75 | 0.57 |
|  |  |  | 2.5 | 0.41 | 0.53 | 0.46 |
|  |  |  | 5 | 0.32 | 0.41 | 0.52 |
|  |  |  | 10 | 0.36 | 0.49 | 0.46 |
|  |  |  | 20 | 0.26 | 0.31 | 0.37 |
|  |  |  | 50 | 0.51 | 0.51 | 0.52 |
|  | 4’-F-CBD | T25 | 1.25 | 0.21 | 0.24 | 0.53 |
|  |  |  | 2.5 | 0.26 | 0.20 | 0.53 |
|  |  |  | 5 | 0.27 | 0.23 | 0.56 |
|  |  |  | 10 | 0.32 | 0.19 | 0.62 |
|  |  |  | 20 | 0.33 | 0.29 | 0.68 |
|  |  |  | 50 | 0.52 | 0.52 | 0.79 |

**Supplementary information 3**

3D OrbiSIMS of U373-V cells exposed to CBD for 3, 6, 24 and 72 h. Peak intensity (secondary ion counts) normalised to the TIC, with deviation below (ppm). Average of n = 3 technical repeats. One-way ANOVA performed, α = 0.05, * = p<0.05 to compare to the control.

| Sample |  | Me-Guanine | Me-Cytosine | Me-Adenine | Me-Thymine |
| --- | --- | --- | --- | --- | --- |
|  | **Formula** | C_6_H_6_N_5_O^-^ | C_5_H_6_N_3_O^-^ | C_6_H_6_N_5_^-^ | C_5_H_5_N_2_O_2_^-^ |
|  | **m/z** | 164.0577 | 124.0516 | 148.0628 | 125.0357 |
| Control | | 1.42 ×10^-5^  ± 9.29 ×10^-6^ | 6.47 ×10^-6^  ± 2.02 ×10^-6^ | 1.94 ×10^-5^  ± 8.28 ×10^-6^ | 3.09 ×10^-4^  ± 4.99 ×10^-5^ |
|  |  | 0.3 | -0.2 | 0.1 | 0.1 |
| CBD 3 h | | 0 | 3.19 ×10^-5^  ± 2.36 ×10^-5^ | 0 | 4.96 ×10^-4^  ± 3.95 ×10^-4^ |
|  |  | - | -0.4 | - | -0.3 |
| CBD 6 h | | 0 | 0 | 0 | 7.86 ×10^-6^  ± 4.68 ×10^-6^ |
|  |  | - | - | - | 0.3 |
| CBD 24 h | | 3.16 ×10^-5^  ± 3.26 ×10^-6^  * | 4.79 ×10^-5^  ± 1.03 ×10^-5^  * | 4.77 ×10^-5^  ± 3.92 ×10^-6^  * | 1.32 ×10^-3^  ± 2.97 ×10^-4^  * |
|  |  | 0.2 | -0.2 | 0.2 | -0.1 |
| CBD 72 h | | 0 | 0 | 0 | 6.69 ×10^-6^  ± 1.44 ×10^-6^ |
|  |  | - | - | - | -0.4 |

**Supplementary information 4**

Principal component analysis of OrbiSIMS data of U373-V cells exposed to CBD or CBD and imidazotetrazine compounds, compared to controls. Principal components showing the largest difference between the control sample (orange) and dosed sample (blue) are highlighted. Key fatty acids were identified; mass (m/z) and peak deviation (ppm) are shown.


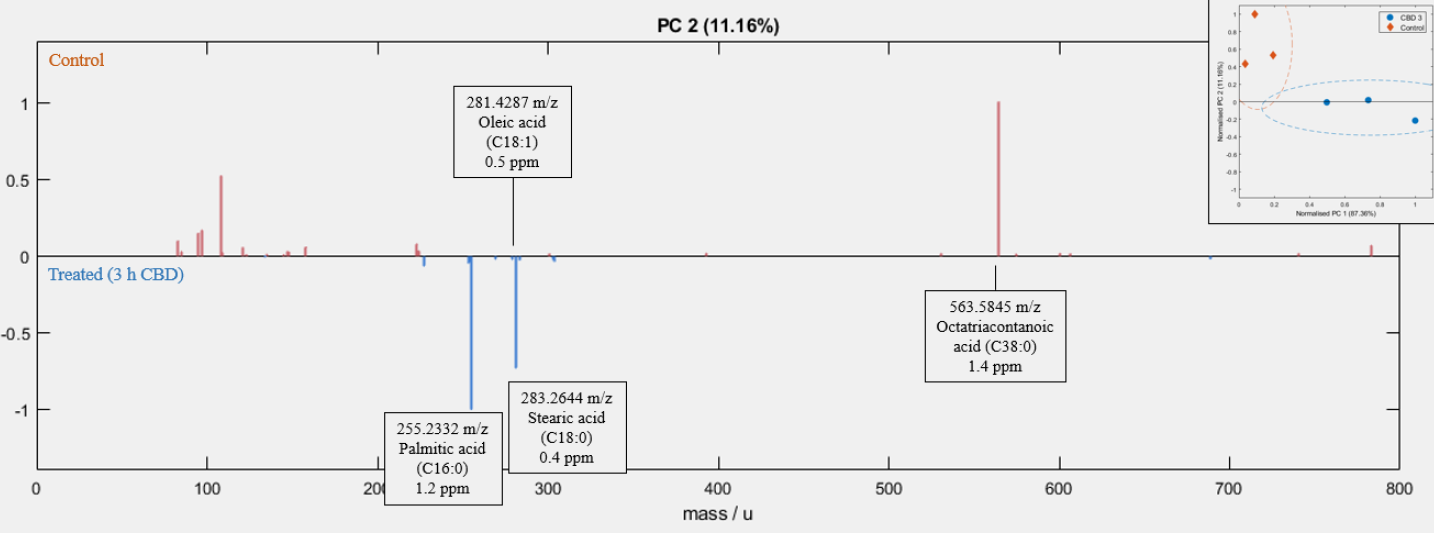


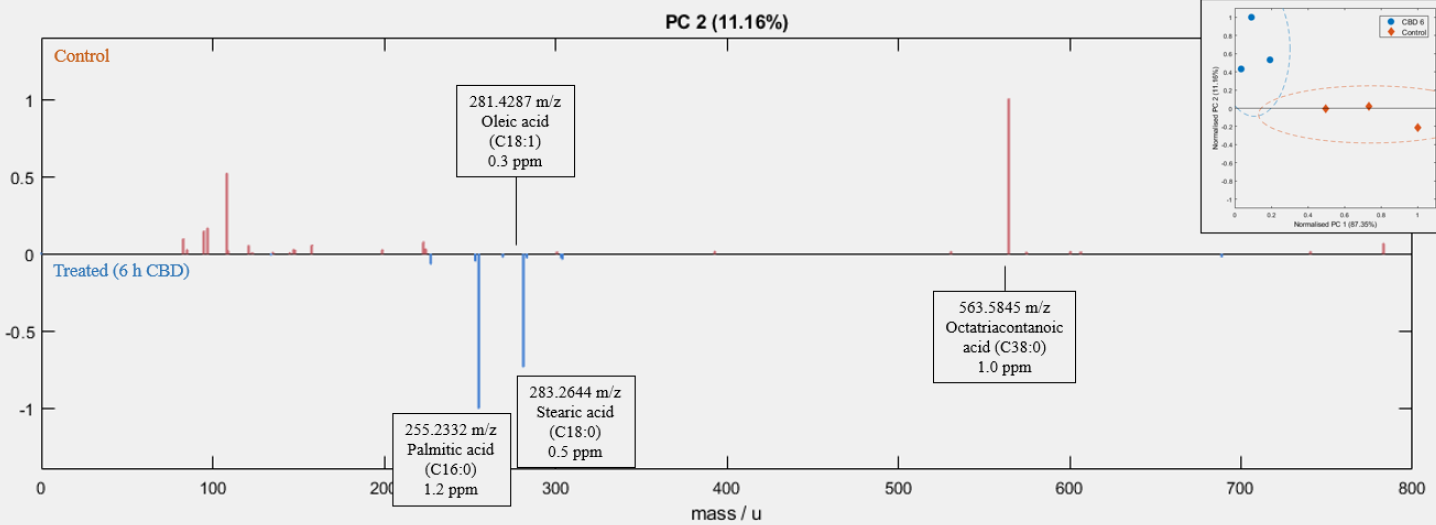


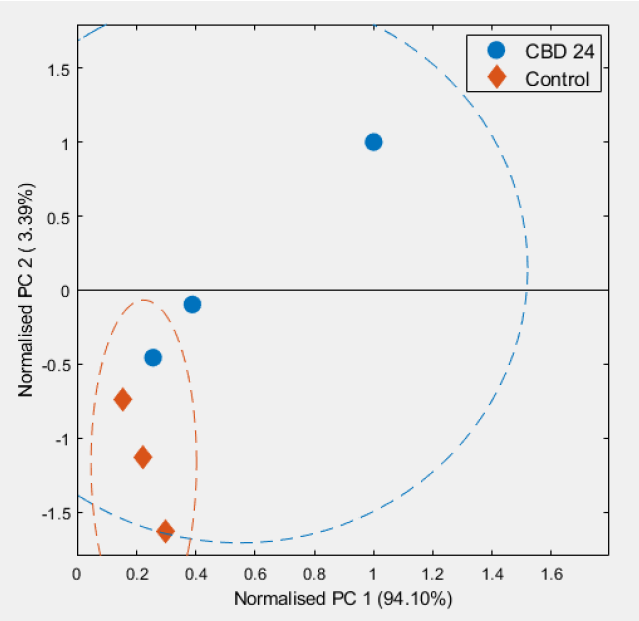


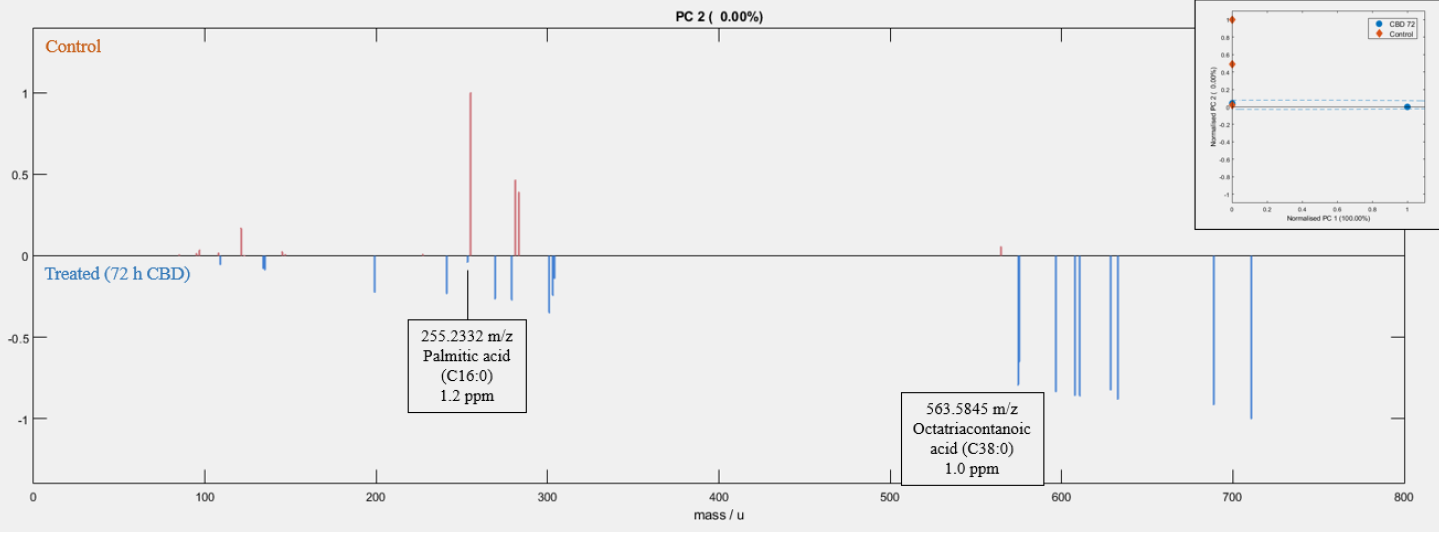


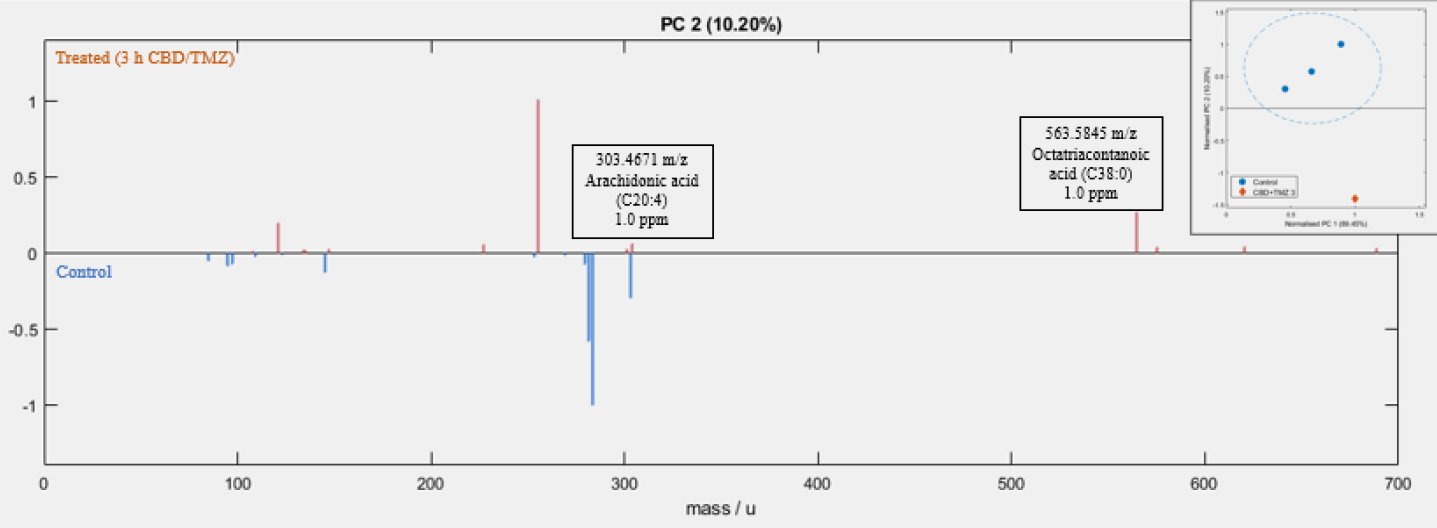


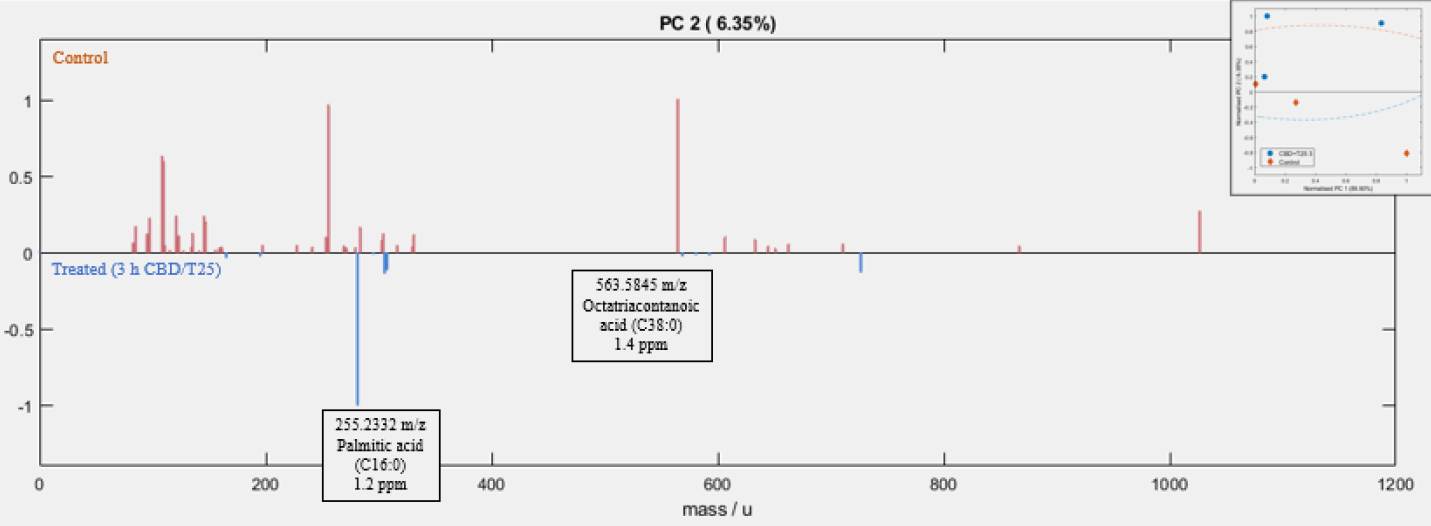

Supplement: Supplementary file 1 — Supplementary information [file 44276_2024_88_MOESM1_ESM.docx]
